# CK20 and CK5/6 Immunohistochemical Staining of Urothelial Neoplasms: A Perspective

**DOI:** 10.1155/2020/4920236

**Published:** 2020-11-04

**Authors:** Mohammed Akhtar, Sameera Rashid, Mohamed Ben Gashir, Noheir Mostafa Taha, Issam Al Bozom

**Affiliations:** Department of Laboratory Medicine and Pathology, Hamad Medical Corporation, Doha, Qatar

## Abstract

Cytokeratins belong to the family of intermediate filaments. They are expressed in a highly specific manner in epithelial cells where they play a crucial role in the integrity and mechanical stability of the cells. Several types of cytokeratins have been described in normal as well as neoplastic urothelium. In the case of urothelial neoplasms expression of CK20 and CK5/6 has been shown in several studies to have diagnostic and prognostic implications. Thus, low-grade urothelial carcinoma manifests CK expression limited to the umbrella cells, while high-grade tumors usually have an expression in the entire thickness of the urothelium except for the basal layer. CK5/6 expression on the other hand is expressed in the basal cells in all low-grade and some high-grade urothelial carcinomas. Diffuse CK20 staining accompanied by loss of CK5/6-positive basal layer is usually associated with aggressive clinical behavior. Double staining of the slides for these cytokeratins may facilitate proper interpretation and correlation.

## 1. Introduction

Cytokeratins belong to the family of 10 nm intermediate filament cytoskeleton present in all the cells. Intermediate filament proteins are expressed in a highly cell type-specific manner, and herein keratins represent the typical intermediate filament category present in epithelial cells. These filaments are a complex group of water-insoluble polypeptides, ranging in size from 40,000 to 68,000 Mr. In epithelia, keratin filaments may be bundled as tonofilaments, braid the nucleus, span through the cytoplasm, and attach to the cytoplasmic plaques of the typical epithelial cell-cell junctions, the desmosomes. Thus, keratins play a major functional role in the integrity and mechanical stability of both the single epithelial cells and that of the epithelial tissues and are an inherent part of the continuum of stability from the single cell to the tissue formation [1–6].

The keratin proteins may be divided into small acidic type I and large basic to neutral type II keratins. A unique property of the keratins is that, in contrast to the other intermediate filament proteins, they only can constitute their filamentous stage by pair formation of type I and type II (1 : 1) molecules. Among human keratins, the recent consensus nomenclature comprises the type I keratins K9–K10, K12–K28, and K31–K40 and type II keratins K1–K8 and K71–K86. In the human genome, the keratin genes are clustered at two different chromosomal sites: chromosome 17q21.2 (type I keratins, except K18) and chromosome 12q13.13 (type II keratins and K18). It is thought that the two cytokeratin subfamilies arose through gene duplication and subsequent divergence of the CK8 and CK18 [2–6].

Each type of epithelial cell expresses a characteristic combination of two to ten cytokeratin isotypes. Thus, normal epithelial cells in vivo or in vitro can be identified with respect to their cytokeratin isotype profile. The characteristic cytokeratin profiles of the different normal epithelial cell types tend to be retained following neoplastic transformation and this feature may be exploited in tumor diagnosis. However, cytokeratin profiles of epithelial cells may also reflect functional differentiation, rather than tissue of origin. Thus, metaplastic cells express cytokeratin profile characteristics of their morphology rather than that of their normal counterpart [1].

## 2. Cytokeratin Expression in Urothelium during Embryologic Development

The cytokeratins are the first intermediate filament type to appear during embryogenesis, and all cells at some stage of fetal development are cytokeratin positive. The first cytokeratin isotypes to be expressed are the CK8 + CK18 pair. During embryological epithelial-mesenchymal transformation, cytokeratin expression is lost by certain lineages of cells, which either remain cytokeratin negative and develop into connective tissue or later re-express cytokeratins of secondary epithelia [1]. Embryologic development of the urinary bladder is from the cloaca, a hindgut structure, that is a common chamber for gastrointestinal and urinary waste. In the 4th–7th weeks of development, the cloaca is divided by the urorectal septum into two parts: posterior anorectal canal and anterior urogenital sinus. The upper part of the urogenital sinus forms the urinary bladder [7].

During the early development of the bladder, the lining epithelium gradually transforms from simple endodermal epithelium to multilayered urothelial lining. As the pattern of the endodermal lining in the GI tract, the developing urothelial lining is initially diffusely positive for CK20 in all layers. During subsequent maturation of the urothelium, a basal layer develops which may be demonstrated by CK5 and CK13 positivity. Further maturation of urothelium involves loss of CK20 in virtually all layers except for the umbrella cells. Thus, there appear to be three distinct phases of urothelial development. The first stage is characterized by a lack of basal cells and CK20 expression in all layers. The second phase is determined by the appearance of the basal layer, but the CK20 expression pattern is still diffuse. In the third and final stages, there is maturation of the CK20 expression pattern which is characterized by CK20 only in the umbrella layer. The basal layer is well developed and can be demonstrated by CK5/6 or CK13 immunostaining [8, 9].

## 3. Cytokeratin Expression in Normal Urothelium

The urinary bladder is lined by urothelium which is specialized to function as a barrier to urine and to accommodate changes in the intraluminal volume. Urothelium appears as a multilayered epithelium, with three cell zones: (1) a basal cell layer composed of cells in contact with and orientated perpendicularly to the plane of the basement membrane; (2) the intermediate cell zone composed of 2–5 cell layers; (3) a luminal or superficial cell layer composed of late intermediate cells and large, frequently binucleated, “umbrella” cells with their apical surface facing the lumen. The umbrella cells are characterized by a zonula occludens type of cell junctions and by the presence of specialized plaques of the asymmetric unit membrane in the apical cell membrane and within intracellular fusiform vesicles [10]. This appearance is due to the presence of abundant uroplakin within the cell membrane [11].

**Figure 1:**
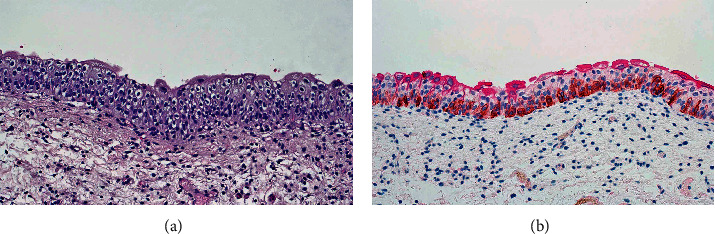
(a) Normal urothelium revealing multiple layers of cells including basal cell, intermediate cells, and umbrella cells. (b) Immunohistochemical stain revealing normal staining pattern of the urothelium. The umbrella cells are decorated by CK20 (magenta). The intermediate cells are unstained. Basal cells are decorated by CK5/6 staining (brown).

In the normal adult urothelium, CK7, CK8, CK18, and CK19 are expressed throughout all urothelial cell layers, CK5 is basally expressed, and CK20 is associated with umbrella cells (Figure 1).

There may also be a few CK20-positive cells in the intermediate cell layer of some normal specimens [1].

## 4. Cytokeratin Expression in Papillary Urothelial Neoplasia

Neoplastic transformation of urothelial cells gives rise to urothelial carcinoma, the commonest form of bladder cancer in most of the countries. Many patients present with superficial tumors which are either noninvasive (pTa) or invade the lamina propria only (pT1). Whilst recurrence is common (50–70%), the disease can usually be controlled by local treatment. Nevertheless, 10–15% of patients with superficial disease eventually progress to muscle-invasive and metastatic disease. An increase in grade, characterized by a loss of differentiation, and the presence of dysplastic changes are both determinants of poor prognosis. In addition, some urothelial carcinomas show basal/squamous differentiation, which has been associated with poorer prognosis [12–14].

**Figure 2:**
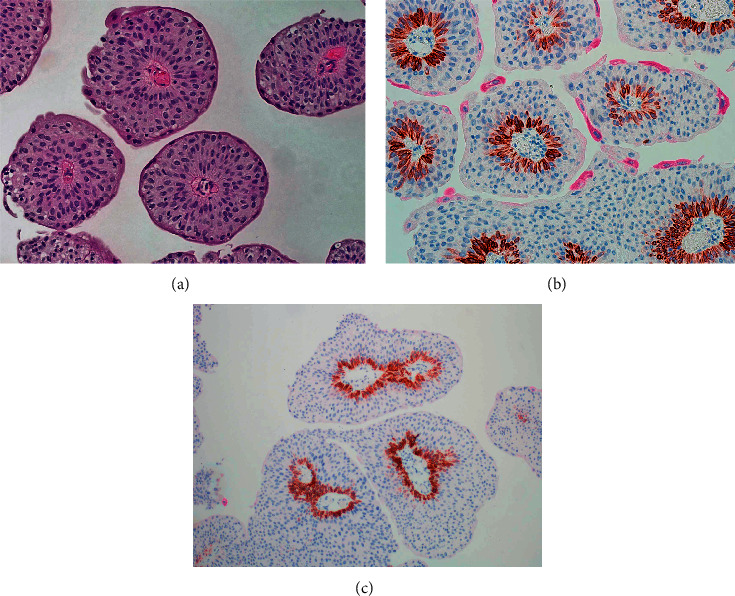
(a) Low-grade papillary urothelial carcinoma. (b) Immunohistochemical stain depicting the CK20 (magenta) expression limited to the umbrella cells and a well-formed basal layer depicted by CK5/6 staining (brown). (c) Low-grade urothelial carcinoma with staining of basal cells but lacking umbrella cells.

The immunohistochemical staining pattern for cytokeratin20 and CK5/6 varies with the type and nature of the urothelial neoplasm. For example, papillary urothelial neoplasms of limited malignant potential (PUNLMP) as well as low-grade papillary carcinomas reveal CK20 limited to the umbrella cells and CK5/6 staining confined to the basal layer, identical to the distribution of these keratins in the normal urothelium (Figure 2).

**Figure 3:**
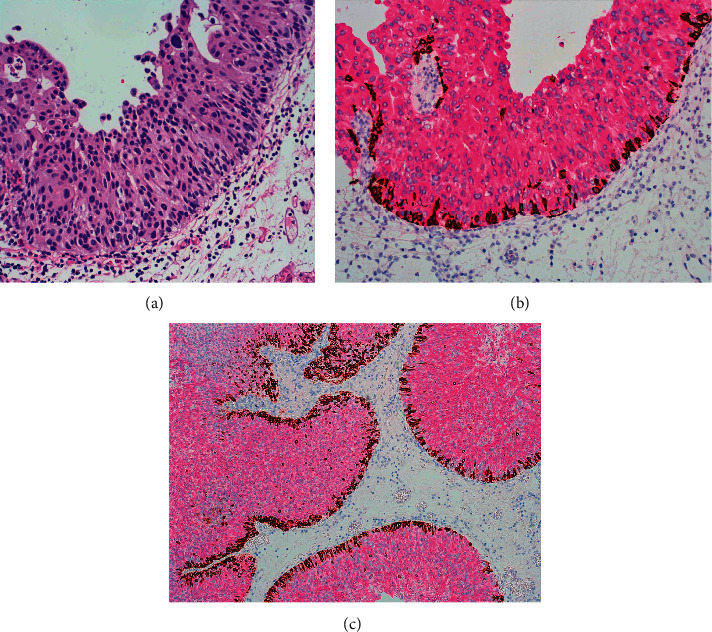
(a) High-grade papillary urothelial carcinoma. (b) High-grade papillary urothelial carcinoma with diffuse full-thickness staining for CK20 and presence of basal layer as demonstrated by CK5/6 staining. (c) High-grade urothelial carcinoma revealing the diffuse presence of basal cells and full-thickness staining for CK20.

High-grade urothelial carcinomas, on the other hand, tend to have diffuse immunoreactivity for CK20 involving the umbrella cells as well as the intermediate layer. In addition, the basal layer may be demonstrated by CK5/6 positivity (Figure 3).

**Figure 4:**
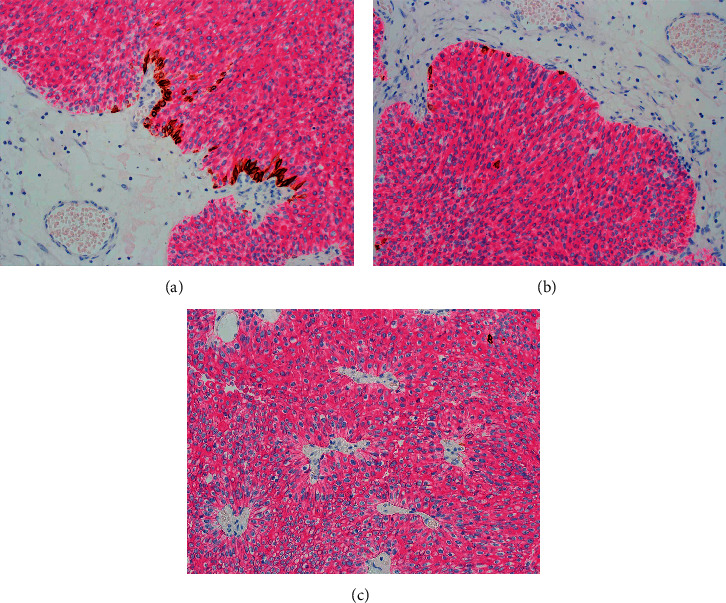
(a) High-grade urothelial carcinoma with partial loss of basal cells but diffuse staining for CK20. (b) High-grade urothelial carcinoma with almost complete loss of basal cells. (c) High-grade papillary urothelial carcinoma revealing diffuse immunohistochemical staining for CK20 and complete absence of basal layer with no staining for CK5/6.

Higher grade papillary urothelial carcinoma, on the other hand, reveals CK20 expression in the entire thickness of the urothelium with the absence of basal layer and negative staining for CK5/6 (Figure 4).

**Figure 5:**
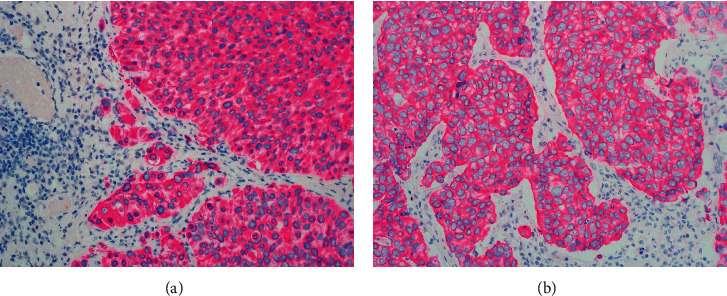
(a) High-grade urothelial carcinoma with early invasion showing strong CK20 cytoplasmic staining and absence of basal cells. (b) High-grade urothelial carcinoma with extensive invasion. The carcinoma cells are diffusely positive for CK20, but the basal layer is absent.

This pattern with diffuse full-thickness CK20 staining in the absence of basal layer staining is also a feature encountered in high-grade urothelial carcinomas with lamina propria or muscle invasion [15–18] (Figure 5).

**Figure 6:**
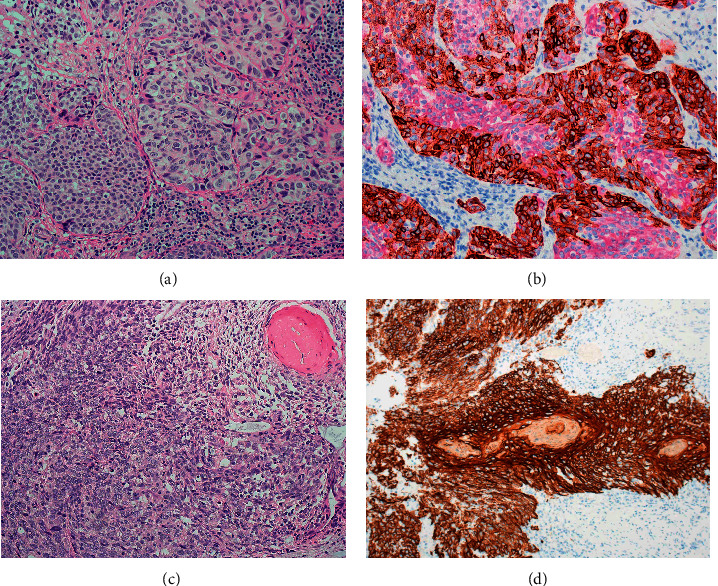
(a) High-grade urothelial carcinoma with focal squamous differentiation. (b) Immunohistochemical staining demonstrating strong CK5/6 staining in the area of squamous differentiation. The remaining part of carcinoma stains for CK20. (c) High-grade urothelial carcinoma with basosquamous morphologic features. (d) Immunohistochemical staining in basosquamous carcinoma featuring strong immunostaining for CK5/6 and complete absence of CK20 positivity.

It has been suggested that the pattern of CK20 staining is a useful adjunct to morphology in the diagnosis of urothelial carcinoma. It has also been suggested that CK20 expression can predict malignant potential in low-grade urothelial tumors, and, therefore, CK20 can be useful in defining treatment strategies for patients with these tumors. CK20 expression was retained by most of the pure urothelial carcinomas, retaining the normal superficial localization pattern in some well-differentiated papillary tumors or showing a heterogeneous or uniformly positive reaction throughout all cell layers. Portions of the carcinoma with squamous differentiation reveal a reduction of CK20 expression. These areas, however, react positively for CK5/6 (Figures 6(a) and 6(b)) in a pattern that is different from the basal distribution of the basal layer [19–22].

In some cases, the entire carcinoma may show basosquamous features. These cases show strong reactivity for CK5/6 with little or no positivity for CK20 (Figures 6(c) and 6(d)).

Two recently published studies have provided additional evidence regarding the clinical significance of various patterns of immunostaining for CK20 and CK5/6. In one of these studies, tumor samples from 222 patients with upper tract urothelial carcinomas who were treated with radical nephroureterectomy were analyzed for the expression of seven basal/luminal immunohistochemical markers (CK5, EGFR, CD44, CK20, p63, GATA3, and FOXA). By using CK5 and CK20, they defined four subtypes of upper tract urothelial carcinomas:

- Exclusively CK20 positive and CK5 negative (CK20+/CK5−)
- Exclusively CK5 positive and CK20 negative (CK20−/CK5+)
- Both markers positive (CK20+/CK5+)
- Both markers negative (CK20−/CK5−)

In multivariate Cox's regression analysis, the CK20+/CK5− subtype was an independent negative prognostic marker with a 3.83-fold increased risk of cancer-specific death (*p*=0.02) compared to the other three subtypes [23].

In a second study, immunohistochemical staining for CK5/6 and CK20 was reported to be correlated with the prognosis of early urothelial carcinoma. In addition, the gene expression profiles of subgroups of non-muscle-invasive papillary high-grade upper tract urothelial carcinoma (UTUC) classified by CK5/6 and CK20 expression levels were studied and correlated with clinical outcomes. These subgroups included CK5/6-high/CK20-low, CK5/6-high/CK20-high, and CK5/6-low/CK20-high. The latter group characterized by low expression of CK5/6 and high expression of CK20 was predictive of worse prognosis of non-muscle-invasive papillary high-grade urothelial carcinomas. Transcriptional analysis revealed 308 differentially expressed genes across the subgroups. Functional analyses of the genes identified cell adhesion as a common process differentially enriched in this subgroup compared to the others, which could explain its high-risk phenotype. Late cell cycle/proliferation signatures were also enriched in this subgroup [24]. Thus, it seems that urothelial tumors characterized by diffuse staining for CK20 in the absence of demonstrable basal layer represent the most aggressive subtype of papillary urothelial carcinoma.

Cytokeratin 5/6 is present in the normal keratinizing epidermis and squamous mucosal epithelium, as well as in basal cells or myoepithelial cells of the breast, salivary glands, and prostate [25–28]. In prostate and breast biopsies, CK5/6 decorates the basal cell layer and thus is useful in the diagnosis of invasive carcinoma, the latter being devoid of basal cells. The architecture of urothelium is different from that of the prostatic or mammary ductal epithelium with the result that the presence or absence of basal cells cannot be used to stratify urothelial neoplasms into benign or malignant types. However, the absence of basal cells in the urothelial neoplasms may indicate poor prognosis. Thus, a high-grade urothelial carcinoma with a preserved basal layer may behave less aggressively as compared to another high-grade tumor in which the basal layer has been lost. In our laboratory, we use double staining for these cytokeratins which is extremely helpful in the proper interpretation of these stains.

**Figure 7:**
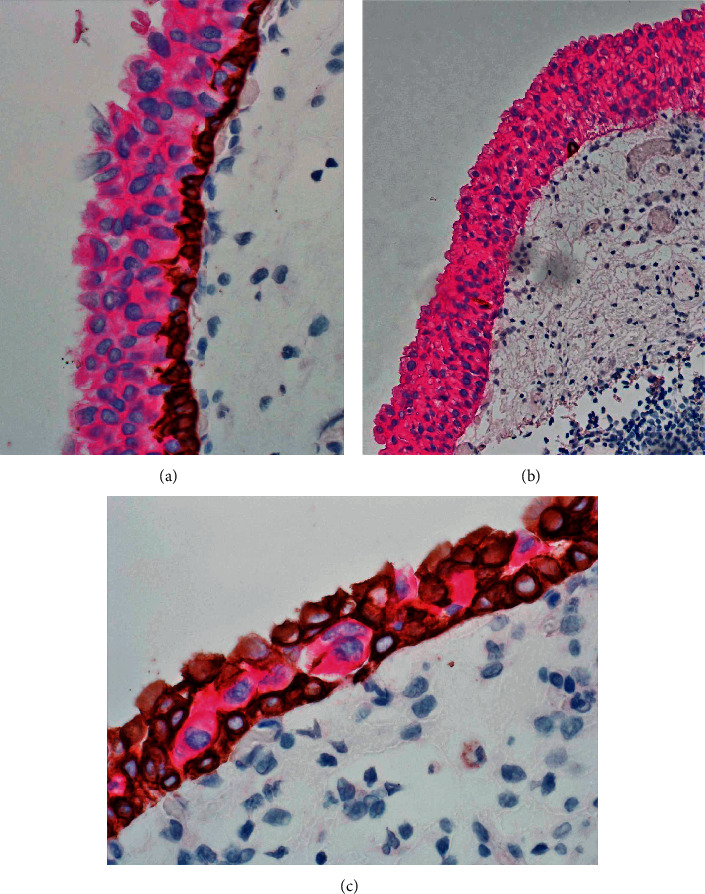
(a) Urothelial carcinoma in situ with strong full-thickness immunostaining for CK20 with a distinct basal cell layer positive for CK5/6. (b) Another urothelial carcinoma in situ featuring strong staining for CK20 and absence of basal layer. (c) Pagetoid extension of urothelial carcinoma demonstrated by strong cytoplasmic staining for CK20 in a background of multiple layers of basal cells reactive for CK5/6.

A similar staining pattern may also be seen in urothelial carcinoma in situ [29, 30]. These lesions are usually strongly reactive for CK20 and may or may not retain the CK5/6-positive basal cell layer. Clinging type of urothelial carcinoma in situ and pagetoid extension of carcinoma cells may also be recognized by positive CK20 staining (Figure 7).

In a minority of cases, carcinoma in situ may be reactive for CK5/6 indicating a basaloid differentiation [31].

## 5. Embryologic Urothelial Differentiation versus Dedifferentiation in Urothelial Neoplasia

**Figure 8:**
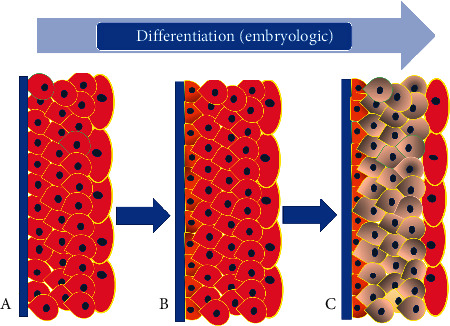
(a) Cartoon depicting stages of differentiation during the embryologic development of the urothelium. During the early stages, the developing urothelium retains CK20 expression normally seen in the cloaca. During this stage, no basal layer is present. (b) During the development of urothelium, the basal layer cannot be demonstrated by staining for CK5/6. (c) Final stage of urothelial differentiation with normal expression of CK20 limited to the umbrella cells and presence of normal basal layer manifesting expression of CK5/6.

**Figure 9:**
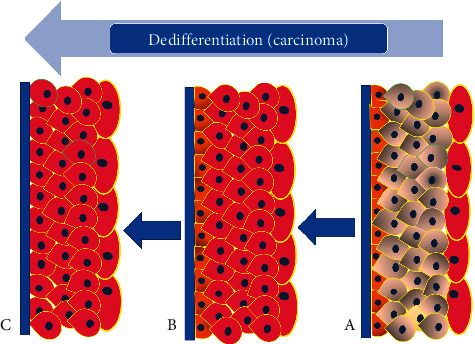
(a) Cartoon depicting immunohistochemical staining pattern in a low-grade papillary urothelial carcinoma with CK20 expression limited to the umbrella cells and presence of a well-defined basal layer which expresses CK5/6. (b) High-grade urothelial carcinoma reveals expression of CK20 in all layers of urothelium except for the basal layer which contains CK5/6. This may represent an intermediate stage of dedifferentiation of the tumor. (c) In high-grade urothelial carcinoma, the basal layer may be absent but the diffuse CK20 pattern of staining is maintained. These features may indicate further dedifferentiation of the carcinoma.

The close correlation between cytokeratin isotype expression patterns and epithelial differentiation suggests that cytokeratins are crucial to epithelial tissue structure and/or function. However, it has been difficult to establish what the precise contributions of individuals or combinations of cytokeratin isotypes are to normal and neoplastic epithelial cell phenotype and behavior. Thus, the changes in CK20 immunolocalization which indicate a higher risk of urothelial carcinoma recurrence probably reflect a secondary change related to dysregulation of the urothelial differentiation program during carcinogenesis rather than indicating that CK20 is directly involved. It may be speculated that the patterns of expression of CK20 and CK5/6 in various urothelial carcinomas tend to mimic the stages of embryologic development and differentiation of urothelium. Thus, high-grade urothelial carcinomas (with or without invasion) which are characterized by diffuse CK20 expression without any basal layer may represent the early stage of urothelial differentiation after the urogenital sinus separates from the cloaca [7–9]. During this stage of development, urothelium manifests diffuse CK20 staining but lacks a basal layer. In some of the high-grade urothelial carcinomas, there is uniform staining of all the layers by CK20, but there is also a well-formed basal layer demonstrable by expression of CK5/6. This may be equivalent to the intermediate stage of embryonic urothelial differentiation. The well-differentiated urothelial carcinomas are characterized by the presence of a mature pattern of CK20 expression where its expression is mostly limited to the umbrella cells, while well-defined basal cell layer immunoreactive for CK5/6 is also present (Figures 8 and 9).

This pattern may be equivalent to the final stage of urothelial development and maturation during the intrauterine period.

In summary, immunohistochemical staining of urothelial carcinoma for CK20 and CK5/6 may provide useful information regarding the nature of urothelial carcinoma and its clinical behavior.

## Data Availability

No data were used to support this study.
